# Associations of meal patterning, dietary quality and diversity with anemia and overweight-obesity among Indonesian school-going adolescent girls in West Java

**DOI:** 10.1371/journal.pone.0231519

**Published:** 2020-04-23

**Authors:** Rina Agustina, Khairun Nadiya, El A. Andini, Ainanur A. Setianingsih, Arini A. Sadariskar, Erfi Prafiantini, Fadila Wirawan, Elvina Karyadi, Manoj K. Raut

**Affiliations:** 1 Department of Nutrition, Faculty of Medicine, Universitas Indonesia–Dr. Cipto Mangunkusumo General Hospital, Jakarta, Indonesia; 2 Human Nutrition Research Center, Indonesian Medical Education and Research Institute (HNRC IMERI), Faculty of Medicine, Universitas Indonesia, Jakarta, Indonesia; 3 Medical Study Program, Faculty of Medicine, Universitas Indonesia–Dr. Cipto Mangunkusumo General Hospital, Jakarta, Indonesia; 4 Former Nutrition International, Country Office Jakarta, Jakarta, Indonesia; 5 Nutrition International, Asia Regional Office, New Delhi, India; Addis Ababa University School of Public Health, ETHIOPIA

## Abstract

**Background:**

Poor diet is a risk factor for anemia, overweight, and obesity among adolescent girls. However, comprehensive assessment on dietary quality and habits in this population is limited. We assessed the association of meal patterning, dietary quality, and dietary diversity with both anemia and overweight-obesity.

**Methods:**

We conducted a cross-sectional survey in 335 school-going adolescent girls aged 12–19 years from three districts in West Java using multi-stage cluster sampling. Meal patterning, Dietary Quality Index for Adolescents (DQI-A), and Dietary Diversity Score (DDS) were determined using 2-day 24-h recall.

**Results:**

Of the girls, 45% were anemic and 17% overweight or obese. Eating occasions of 3–4 times (AOR 2.68, 95% CI 1.21–5.98) and >4 times (AOR 2.43, 95% CI 1.01–5.83) were associated with greater odds of developing anemia compared to eating occasions of <3 times. Adolescent girls who skipped dinner had greater odds of being overweight or obese (AOR 2.13, 95% CI 1.10–4.10) and were less likely to be anemic (AOR 0.56, 95%CI 0.33–0.95) compared to those who did not skip dinner. Difference in energy intake was found between girls who had dinner and skipped dinner (p = 0.05). Mean total DQI-A score was 44.4% ± 7.71% and DDS was 4.0 out of 9.0. DQI-A score was significantly higher in non-anemic compared to anemic girls. Moreover, each unit increment of 1% of total DQI-A score was associated with a 3.967 g/dL increases of hemoglobin after adjustment for confounders. We found differences in total DQI-A score between normal-weight and overweight or obese girls. DDS score was not significantly different between groups, although lower meat, chicken, and fish consumption were correlated with anemia (p<0.01).

**Conclusions:**

Overall, the girls had poor dietary quality and diversity. The findings therefore indicated the importance of improving dietary quality and diversity in a regular meal pattern, especially meal frequency and meal skipping, to reduce the risk of anemia and overweight-obesity among adolescent girls.

## Introduction

Anemia and malnutrition continue to be global health problems, especially among females in developing countries, including Indonesia [1, 2]. Anemia burden counts for 1.6 billion people or 25% of the population worldwide [3]. Meanwhile, 15% of adolescent girls worldwide suffer from anemia, with 6% in developed and 27% in developing countries. According to the Indonesian national health research, the prevalence of anemia in Indonesia was 11.9% in 2007 and had increased to 21.7% in 2013 [2, 4]. A few studies in Indonesia reported that the burden of adolescent anemia ranged between 18–26. However, another study reported an even higher value greater than 50% [2, 5, 6]. The most common cause of anemia was iron deficiency (IDA), and non-nutritional causes included disease-related causes and genetic disorders. However, IDA and non-nutritional anemia are frequently viewed as equal due to the significant role of iron in both conditions [7]. Adolescent girls have a high risk of developing anemia due to low iron intake and absorption, blood loss during menstruation, and increased need for iron to support rapid growth [8, 9]. Being anemic can cause low productivity, decreased cognitive function and development, and higher vulnerability to infection [10].

Malnutrition among adolescent girls is also prevalent in developing countries, including countries in Southeast Asia (24–47%). The high prevalence of adolescent thinness in Indonesia remains a problem followed by an increasing burden of overweight and obesity (2). In 2018, the prevalence of overweight and obesity reached 16% for 13–15 years old, and 13.5% for 16–18 years old [11]. These numbers increased quite dramatically compared to the prevalence of 10.8% for 13–15 years old and 7.3% for 16–18 years old in 2013 [12]. Adolescence is considered a nutritionally important period as it marks rapid growth and development as well as changes in food habits and lifestyle, which requires a high amount of nutrition and energy [13–15]. On the other hand, overweight and obesity in adolescents are associated with an increased risk of non-communicable diseases in child-bearing age and later life [16].

Meal patterning is defined as an individual’s frequency, skipping, and spacing of eating occasions [17]. Aside from meal frequency and quantity, adolescents’ diet quality and diversity are other important variables. Good dietary quality and diversity are needed for growth, especially during adolescence, to prevent macro- and micronutrient inadequacy, which are associated with nutritional status problems [18–21]. At the same time, meal patterning together with control of dietary quality and diversity are important in managing diet strategies to prevent overweight and obesity in adolescents [22].

Diet quantity, as well as quality, depends on cultural tradition, social and economic status, food practices, financial allocation, and choices [15]. Some factors are known to be associated with adolescence, such as fast food and fortified food availability, social media, sociocultural factors, available time for meal, and economic status [15]. To look for the adequacy of nutritional intake is to assess the quality and the diversity of the diet consumed. Several approaches are available to assess nutrient intake and the quality of diet consumed, including defining what a “meal” is and how it relates to overall nutritional quality. However, studies exploring adolescent diets in a comprehensive manner, from frequency of eating, quality and diversity, as well as the meeting of dietary guidelines and its association with health and nutritional outcomes, are limited. Therefore, we assessed the meal patterning, dietary quality, and dietary diversity among adolescent girls to determine which factors are associated with anemia and overweight-obesity in adolescence. The results of this study can be useful for designing health and nutritional programs, plans, and guidelines targeted towards adolescent girls.

## Materials and methods

### Study design and population

A cross-sectional study was conducted in three districts of West Java province, Indonesia, in 2016 as a part of the baseline survey of ‘Iron Folate Supplementation Program’ conducted by the Department of Nutrition, Faculty of Medicine, Universitas Indonesia for Nutrition International. The subjects were school-going adolescent girls aged 12–19 years. Minimum sample sizes of 240 and 191 were required to detect associations between dietary quality and anemia, as well as, overweight-obesity, with logistic regression analysis (95% confidence interval, 80% power, assumed OR 1.79 for low dietary quality to be anemic [23] and OR 0.55 for high dietary quality to be obese [24]). With a probable non-response rate of 10%, a total sample of 300 was decided. As the current study was a part of a baseline data of the larger intervention study of iron folic acid supplementation, the eventual number of respondents corresponded to this study with a total of 340 adolescent girls. This number was powered to detect a 15% point change between 18.4% assumed anemia prevalence in adolescent girls [12] and an anticipated 3% at endline survey. The analysis was for two sample tests with standard statistical assumptions (two-sided test; alpha = 0.05; 0.8 power, and non-continuity), rounded up to account for endline survey, design effect, and assumed non-response rate. The use of 340 sample enable better power for the current study outcomes.

The sample was selected using the probability proportional to population size method based on cluster selection from the villages of the baseline study of ‘Iron Folate Supplementation Program’. Clusters were selected based on the 2015 population census; 34 villages were selected by adopting a probability proportional to population size method, while households were listed from the chosen cluster to prepare a sampling frame for school-going girls in the 12–19 years age group. Eventually, 17 clusters were selected. The households in the selected clusters were then listed to prepare a sampling frame for the school-going adolescent girls. The clusters were then segmented to 100 households each, which were randomly selected for sampling. The required number of respondents (10 in each cluster) was selected via a systematic random sampling method from the sampling frame of the households. If the desired number of households was not available in the selected segment, an adjacent segment was covered to obtain the required number of respondents. The inclusion criteria were girls aged 12–19 years who attended junior/secondary high school and have already had menarche. All adolescent girls and their parents who were eligible to participate were required to sign an informed assent and consent, respectively. Those who refused to participate, dropped out and/or quit school were excluded from the study. Three hundred and forty school-going adolescent girls were included in the beginning of the survey. However, 5 of them did not complete the 2-repeated 24-h dietary recalls assessments and, for that reason, were excluded, leaving 335 school-going adolescent girls’ data to be included as study participants. Six individuals with severe anemia (Hb ≤ 8) were excluded from analyses that involved anemia and Hb value variables, considering the condition was likely to be affected by other comorbidities and might cause bias in the analysis related to the diet. Meanwhile, nine individuals with an underweight status (BAZ <-2 SD) were excluded from analyses involving BMI status and BAZ variable, because underweight was not considered as normal comparison. Leaving total of 329 participants for anemia and 326 participants for overweight-obesity variables. The study was conducted as a baseline survey; therefore, no intervention had been performed on the adolescent girls at this period.

### Data collection and variables

The data collection methods and tools were pre-tested in two villages which were not selected for the survey area. The pre-test was aimed to look for possible errors in data collection by investigating the time allocation, the respondents’ understanding of the questions, possible confusing keywords, the questioning methods of the interviewer, and whether the portion standardization was suitable. Using pre-tested interviewer-administered household structured questionnaire, trained fieldworkers visited households to collect the socio-demography and dietary intake data.

Trained enumerator collected the socio-demographic data that were included as covariates, namely socio-economic status (status of dwelling unit, type of floor and latrine availability), demographic variable (number of family, type of family, age and education level), and source of health information (frequency of reading newspaper, watching TV, listening to radio and access to internet). Type of floor and latrine availability were included as proxies for socioeconomic and sanitary condition that relates to infection-related anemia. Meanwhile, source of health information were included as variable proxies to health information exposure which are related to anemia prevalence [25, 26].

Data of meal pattern, dietary quality, and dietary diversity as independent variables were assessed by a two-non-consecutive-day 24-hour recall (one on a weekday and one on a weekend day) by a trained nutritionist. We minimized bias in the dietary assessment by taking the following actions: recruiting experienced fieldworkers and nutritionists from diploma or bachelor in nutrition education background, pre-training the recruited fieldworkers and nutritionists, pre-testing the questionnaire and the interviewer, standardizing portions of each meal in the 24-hour recalls using picture guide with colored photographs of different portion sizes, assigning the same person to take the repeated recalls for each subject, and using the Indonesian Food Composition Tables that is recognized by the Food and Agriculture Organization of the United Nations (FAO-UN) [27] to calculate the nutrient contents of local foods and snacks.

The dependent variables were anemia and overweight-obesity status. Anemia status was determined by capillary hemoglobin (Hb) concentration <12 g/dL measured by the HemoCue201^TM^ portable device. HemoCue201^TM^ has a 0.85 sensitivity and 0.94 specificity [28] and has been used in various studies that required Hb measurement [29] The Hb level was adjusted for the altitude of the area, as recommended by WHO [30]. Anthropometric measurements were done for weight and height. Body weight was measured using a digital scale (SECA^TM^) with a precision of 0.05 kg, and height was measured with a short board fixed to the wall, with a precision of 0.1 cm. BMI was calculated by dividing body weight (kg) by height squared (m^2^). Weight status was defined by Body Mass Index (BMI) for age z-score (BAZ) compared to the WHO growth curves (WHO, 2007) and were grouped as overweight/obese (>+1SD) vs non-overweight/obese (≤+1 to ≥ -2 SD).

### Meal patterning, dietary quality, and dietary diversity assessment

The mean intake and mealtime information from the 2-day 24-hour recall results was defined to determine meal patterning, dietary quality, and dietary diversity of the subjects. Meal patterning was determined using a ‘neutral approach’, based on the quantitative information of the number and frequency of their eating occasions, meal spacing, and meal skipping. An eating occasion was considered a meal if it was an event of eating, exclusive of events with only beverages, which occurred more than 15 minutes apart from the previous or next event and with a minimum energy intake of 50 kcal (210 kJ) [17]. Frequency is the total number of eating occasions per day. Breakfast was defined as a meal occurring between 00.00 and 11.00, lunch between 11.00 and 17.00, and dinner between 17.00 and 00.00 [31]. Meals were considered to be skipped when the respondent reported eating no meals during one of these time periods. Spacing was calculated as the mean time between meals [32].

Dietary quality was assessed by the Dietary Quality Index for Adolescent (DQI-A) score, while dietary diversity was assessed by the Dietary Diversity Score (DDS). These two different methods were selected based on the tools’ merit on assessing each assigned component. DQI-A considers the total diet quality in general based on the food’s nutrient, diversity, and equilibrium, with the total DQI-A score as the output. While, DDS is more specific to dietary diversity in population-level compared to DQI-A [33].

The DQI-A score system used in the current study was adapted from the dietary quality scoring tool developed by the Helena study. The tool was already validated for the use in adolescents [34]. To obtain the total DQI-A score, each adolescent’s food intake from the 2-day 24-hour recall was divided into nine recommended food groups, namely 1) water, 2) bread and cereal, 3) potato and grains, 4) vegetables, 5) fruits, 6) dairy products, 7) cheese, 8) protein source food, and 9) oil and fat. The foods were then further categorized into three scoring groups; preference, intermediate, and low-nutrient energy-dense, which carried a weighting factor of +1, 0, and -1 respectively. Based on each food intake quantity and weighting factors, dietary quality (DQ), dietary diversity (DD), dietary adequacy (DA), and dietary excess (DEx) were counted. DQ was measured by first multiplying the consumed amount per food with its weighing factor, summing it up with all the other foods consumed, and then dividing it by the total amount of food consumed. Good dietary quality was reflected by a high dietary quality component score. DD score was counted by adding one point for each food group and then dividing it by nine, which is the total number of DQI-A food groups. Dietary equilibrium (DE) score was obtained from subtracting DEx score from the DA score. All scores were converted to percentages. The overall range of DQI-A score can be from (-33)% to 100%, calculated from the sum of DQ, DD, and DE and then divided by three [34]. A high DQI-A score means good dietary quality. A 100% score means perfect diet quality and diversity with all type of meals within recommended range [34].

DDS is a scoring system used to assess the diversity of all foods consumed during the last 24 hours or during a certain time period based on a simple count of consumed food groups. Because of the different nutritional needs of adolescent girls compared to younger children, we used the category of DDS for reproductive-age woman, so called the Women’s Dietary Diversity Score guide published by the FAO-UN. The tool has been validated to assess micronutrient adequacy in women globally [33]. The food categories consisted of the following groups: 1) starch, 2) green vegetables, 3) vitamin A-rich fruits and vegetables, 4) other fruits and vegetables, 5) organ meat, 6) meat and fish, 7) eggs, 8) legumes, nuts, and seeds, and 9) dairy products [33, 35]. The scores were categorized into low-, medium-, or high-diversity diet based on 0–3, 3.01–6 and 6.01–9 food groups reportedly consumed averaged from the two days of dietary recalls.

### Data analysis

The dietary recall data was analyzed using the Nutrisurvey^TM^ software to obtain the nutritional content of the food consumed by subjects. In case the food was not listed, a new recipe would be added based on the Indonesian Food Composition Table or the manufacturer’s ingredient list for some specific brands. However, when the nutrient content information was absent, other FAO/UN approved food databases, such as Canada’s national food composition database and the United States Department of Agriculture National Nutrient Database, were used [36]. Energy, macronutrient, and micronutrient (iron in particular) contents were included within the components of all of the foods listed in Indonesian Food Composition Table [27], therefore, it was suitable to be used as nutritional content reference for the current study. Energy and iron intake data taken from dietary analysis were also included as possible confounders.

BMI was analyzed using the AnthroPlus-2007^TM^ software for personal computers. Anemia status, overweight-obesity status, DQI-A score, DD score, meal frequency and skipping, and participants’ characteristics were analyzed using the statistical package for social sciences (SPSS^TM^) version 20. Normality of the distribution was tested using the Kolmogorov-Smirnov test. Mean and standard deviation (SD) were presented in normally distributed data, while median and inter-quartile range (IQR) were presented in non-parametric data. Bivariable logistic regression was performed to identify factors associated with anemia and overweight-obesity for each variable and characteristic of respondents. Then, variables observed in the bivariable logistic regression analysis with a p-value < 0.25 were included in the multivariable binary logistic regression as confounding factors. Inter-cluster coefficient (ICC) for anemia, overweight-obesity, Hb, and BAZ score were measured using mixed model analysis. Cluster variable was considered a confounding factor if ICC was > 10%. As the secondary outcome, correlation of the total DQI score with Hb and BAZ score were performed by linear regression as the aforementioned values were measured in determining anemia and overweight-obesity, respectively. The linear regression analysis was done after fulfilling the assumption of normality of distribution. The strength of the statistical association was measured by adjusted odds ratios (AOR) and 95% confidence intervals. Results with p-value < 0.05 were considered statistically significant.

### Ethical considerations

The protocol was submitted for ethical clearance and was approved by the Ethical Committee of the Faculty of Medicine, Universitas Indonesia as the institutional review board through a letter numbered 72/UN2.F1/ETIK/2016. The ethical committee approved the blood sample collection, method and device testing as well as disposal procedures conducted in the field. All eligible participants and their parents were required to sign an informed assent or consent, respectively, which explained the objective of the study, the step-by-step procedure that will be undergone by the participants, and the anonymity and confidentiality of the participant’s information. Anonymity and confidentiality were ensured by omitting the name of the participant and replacing it with a code.

## Results

### Participants’ characteristics

**Table 1** summarizes the participants’ characteristics, including socio-economic status, demographic variable, source of health information, and the association of each characteristic with anemia and weight status. Age, education level, frequency of reading newspaper, frequency of watching TV, access to internet, and use of social media were found to be the potential confounders of anemia. Meanwhile, age and mother’s level of education were inversely associated with overweight-obesity. Listening to the radio was included as a potential confounding factor for overweight-obesity.

**Table 1:** Socioeconomic factors, demographic factors and source of health information in association with anemia and weight status (overweight-obese) among school-going adolescent girls in selected districts of West Java.

Variables	n (%)	Anemia (n = 329)		Overweight-Obese (n = 324)	
		n (%)	OR (95% CI)	n (%)	OR (95% CI)
**Socioeconomic factors**					
** Ownership status of dwelling unit**					
** **Own	278 (83)	121 (44.0)	0.91 (0.51–1.64)	49 (18.3)	1.34 (0.60–3.02)
** **Others^a^	57 (17)	25 (46.3)	1.00	8 (14.3)	1.00
** Type of floor**					
** **Permanent	310 (92.5)	138 (45.2)	1.65 (0.69–3.98)	51 (17.1)	0.65 (0.25–1.71)
** **Others^b^	25 (7.5)	8 (33.3)	1.00	6 (24.0)	1.00
** Availability of latrine**					
** **Own	312 (93.1)	138 (45.1)	1.54 (0.63–3.74)	55 (18.2)	2.23 (0.51–9.81)
** **Others^c^	23 (6.9)	8 (34.8)	1.00	2 (9.1)	1.00
**Demographic factors**					
** Age (years)**^**1**^ ** **12–14 ** **15–19	15 (12–18) 162 (48.4) 173 (51.6)	62 (39) 84 (49.4)	0.65 (0.42–1.01)^+^ 1.00	32 (20.3) 25 (15.1)	1.43 (0.81–2.55)^+^ 1.00
** Family type**					
** **Nuclear family	287 (85.7)	122 (43.1)	0.70 (0.37–1.30) 1.00	51 (18.4)	1.54 (0.62–3.83) 1.00
** **Others^d^	48 (14.3)	24 (52.2)		6 (12.8)	
** Adolescent’s education level**					
** **Junior high school	212 (63.3)	86 (41.1)	0.70 (0.45–1.10)^+^ 1.00	38 (18.4)	1.18 (0.64–2.16) 1.00
** **Senior high school	123 (36.7)	60 (50.0)		19 (16.1)	
** Father’s education level (years)**					
** **≤9	221 (66)	92 (42.8)	0.83 (0.53–1.31) 1.00	35 (16.6)	0.82 (0.46–1.48) 1.00
** **>9	114 (34)	54 (47.4)		22 (19.5)	
** Mother’s length of education (years)**					
** **≤9	246 (73.4)	102 (42.5)	0.76 (0.46–1.23) 1.00	35 (14.8)	0.52 (0.29–0.95)* 1.00
** **>9	89 (26.6)	44 (49.4)		22 (25.0)	
**Source of health information**					
** Frequency of reading newspaper**					
** **Not at all	218 (65.1)	90 (41.5)	0.71 (0.45–1.12) ^+^	38 (18.0)	1.08 (0.59–1.99)
** **Others^e^	117 (34.9)	56 (50.0)	1.00	19 (16.8)	1.00
** Frequency of watching TV**					
** **Almost every day	286 (85.4)	125 (44.5)	1.03 (0.56–1.91)	47 (17)	0.78 (0.36–1.67)
** **Others^f^	49 (14.6)	21 (43.8)	1.00	10 (20.8)	1.00
** Frequency of listening radio**					
** **Not at all	208 (62.1)	97 (46.9)	1.31 (0.84–2.07)	30 (14.9)	0.62 (0.35–1.11)^+^
** **Others^g^	127 (37.9)	49 (40.2)	1.00	27 (22.0)	1.00
** Access to internet**					
** **No	42 (12.5)	14 (34.1)	0.61 (0.31–1.22)^+^	8 (20.0)	1.20 (0.52–2.76)
** **Yes	293 (87.5)	132 (45.8)	1.00	49 (17.3)	1.00
** Use of social media**					
** **No	58 (17.3)	19 (33.3)	0.57 (0.31–1.04)^+^	11 (19.6)	1.18 (0.57–2.45)
** **Yes	277 (82.7)	127 (46.7)	1.00	46 (17.2)	1.00

SD, standard deviation; CI, confidence interval; OR, crude odd ratio

*Significant association when p<0.05 in logistic regression

^+^For confounding factors when p<0.25 in logistic regression

^a^Rental, stay for free, family/non-resident

^b^Semi-permanent: wood, bamboo; non-permanent: soil

^c^Public, river, bush, forest

^d^Extended family/single parent

^e^Almost every day, at least/less than once a week

^f^At least/less than once a week, not at all

^g^Almost every day, at least/less than once a week

^1^Median (25^th^, 75^th^ percentile)

### Prevalence of anemia and overweight-obesity

Overall, the mean and median of the Hb concentration of the adolescent girls (n = 335) were 11.97 (SD = 1.69) and 12.15 g/dL. The mean and median BAZ score were -0.03 (SD = 1.07) and -0.09. The prevalence of anemia in this study was 45.4%. Of the anemic adolescent girls, 23%, 20.6%, and 1.8% had mild, moderate, and severe anemia. Weight status was distributed into thin (3.6%), normal (79.4%), and overweight-obese (17%).

### Meal patterning

The mean frequency of eating occasions was 3.72 ± 1.08 during weekdays and 3.67 ± 1.23 during weekend. Dinner was skipped more frequently (weekends: 25%, weekdays: 23%) than breakfast (7.5% and 4.8%) or lunch (9.3% and 6.3%). The mean spacing between meals per day was 241.6 ± 97.1 minutes on weekends and 255.9 ± 121.9 minutes on weekdays.

**Table 2** represents the association between meal patterning characteristics and anemia using logistic regression. Three to four and >4 times frequency of eating occasions during weekdays and skipping dinner had a significant association with anemia among adolescent girls. Three to four and >4 times eating occasions per day show, respectively, 2.7 (AOR 2.68, 95% CI 1.21–5.98) and 2.4 times (AOR 2.43, 95% CI 1.01–5.83) greater odds to have anemia than those who ate less frequently (<3 times/day). Skipping dinner during weekends was inversely associated with being anemic (OR 0.58, 95% CI = 0.35–0.97 and AOR 0.56, 95% CI = 0.33–0.95).

**Table 2:** Association between meal patterning and anemia among school-going adolescent girls in selected districts of West Java (n = 329).

Meal Patterning	Anemia				
	Yes, n (%)	OR (95% CI)	P value*	AOR (95% CI)^a^	P value*
**Eating Frequency (times/day)**					
** ***Weekday*					
** **<3	10 (26.3)	1.00		1.00	
** **3–4	100 (47.6)	2.55 (1.18–5.51)	0.018*	2.68 (1.21–5.98)	0.016*
** **>4	36 (30.4)	2.24 (0.96–5.21)	0.061	2.43 (1.01–5.83)	0.047*
** ***Weekend*					
** **<3	23 (44.2)	1.00		1.00	
** **3–4	85 (40.7)	0.86 (0.47–1.60)	0.641	0.76 (0.41–1.43)	0.398
** **>4	38 (55.9)	1.60 (0.77–3.30)	0.207	1.83 (0.86–3.91)	0.118
**Spacing between eating (minutes)**					
** ***Weekday*					
** **<180	32 (47.1)	1.00		1.00	
** **180–290	78 (42.4)	0.82 (0.53–1.43)	0.486	0.77 (0.43–1.40)	0.391
** **>290	36 (47.4)	1.01 (0.53–1.95)	0.970	0.97 (0.49–1.94)	0.946
** ***Weekend*					
** **<180	31 (46.3)	1.00		1.00	
** **180–300	87 (46.0)	0.99 (0.57–1.73)	0.973	0.95 (0.53–1.70)	0.862
** **>300	28 (38.4)	0.72 (0.37–1.41)	0.344	0.65 (0.32–1.32)	0.237
**Breakfast Skipping** *Weekday*					
** **No	138 (44.1)	1.00		1.00	
** **Yes	8 (50.0)	1.27 (0.46–3.47)	0.643	1.69 (0.56–5.12)	0.354
** ***Weekend*					
** **No	135 (44.3)	1.00		1.00	
** **Yes	11 (45.8)	1.01 (0.46–2.45)	0.881	1.21 (0.52–2.84)	0.654
**Lunch Skipping**					
** ***Weekday*					
** **No	138 (44.5)	1.00		1.00	
** **Yes	8 (42.1)	0.91 (0.36–2.32)	0.837	1.02 (0.41–2.53)	0.973
** ***Weekend*					
** **No	131 (43.8)	1.00		1.00	
** **Yes	15 (50.0)	1.28 (0.61–2.72)	0.516	1.33 (0.60–2.92)	0.480
**Dinner Skipping**					
** ***Weekday*					
** **No	113 (44.8)	1.00		1.00	
** **Yes	33 (42.9)	0.92 (0.55–1.54)	0.759	0.90 (0.52–1.55)	0.700
** ***Weekend*					
** **No	117 (47.8)	1.00		1.00	
** **Yes	29 (34.5)	0.58 (0.35–0.97)	0.036*	0.57 (0.33–0.97)	0.037*

OR, crude odds ratio; AOR, adjusted odds ratio; CI, confidence interval

^a^ AORs were obtained from a multivariable binary logistic regression model adjusted for age; energy and iron intake; highest education of adolescent girl; frequency of reading newspaper; access to internet; use of social media; cluster (ICC 18.4%)

*Statistically significant at p < 0.05

Results on **Table 3** show that adolescent girls who skipped dinner on weekdays were more likely to be overweight (AOR 2.06, 95% CI 1.07–3.99; p = 0.031,). There were no associations between eating frequency and spacing time.

**Table 3:** Meal patterning as factors associated to weight status among Indonesian school-going adolescent girls in selected districts of West Java (n = 324).

Meal Patterning	Overweight-Obese				
	Yes, n (%)	OR (95% CI)	P value*	AOR (95% CI)^a^	P value*
**Eating Frequency (times/day)**					
** ***Weekday*					
** **<3	8 (21.6)	1.00		1.00	
** **3–4	35 (17.0)	0.75 (0.32–1.77)	0.506	0.64 (0.26–1.56)	0.325
** **>4	14 (17.3)	0.75 (0.28–1.97)	0.555	0.69 (0.25–1.85)	0.457
** ***Weekend*					
** **<3	11 (20.4)	1.00		1.00	
** **3–4	38 (18.7)	0.90 (0.43–1.91)	0.784	0.94 (0.43–2.07)	0.876
** **>4	8 (11.9)	0.53 (0.20–1.43)	0.210	0.51 (0.18–1.43)	0.200
**Spacing between eating (minutes)**					
** ***Weekday*					
** **<180	16 (23.9)	1.00		1.00	
** **180–290	31 (17.1)	0.66 (0.33–1.30)	0.230	0.61 (0.30–1.22)	0.162
** **>290	10 (13.2)	0.48 (0.20–1.15)	0.101	0.45 (0.19–1.10)	0.080
** ***Weekend*					
** **<180	8 (11.9)	1.00		1.00	
** **180–300	34 (18.3)	1.65 (0.72–3.77)	0.235	1.67 (0.72–3.87)	0.232
** **>300	15 (21.1)	1.98 (0.77–5.02)	0.153	2.13(0.81–5.58)	0.125
**Breakfast Skipping** *Weekday*					
** **No	54 (17.5)	1.00		1.00	
** **Yes	3 (20.0)	1.18 (0.32–4.33)	0.802	1.35 (0.35–5.16)	0.663
** ***Weekend*					
** No**	54 (18.1)	1.00		1.00	
** Yes**	3 (12.0)	0.62 (0.18–2.14)	0.449	0.55 (0.151.94)	0.348
**Lunch Skipping**					
** ***Weekday*					
** **No	53 (17.5)	1.00		1.00	
** **Yes	4 (19.0)	1.11 (0.36–3.43)	0.856	0.98 (0.31–3.11)	0.978
** ***Weekend*					
** **No	54 (18.3)	1.00		1.00	
** **Yes	3 (10.3)	0.52 (0.15–1.76)	0.291	0.63 (0.18–2.18)	0.460
**Dinner Skipping**					
** ***Weekday*					
** **No	38 (15.3)	1.00		1.00	
** **Yes	19 (25.3)	1.88 (1.01–3.52)	0.047*	2.06 (1.07–3.99)	0.031*
** ***Weekend*					
** **No	40 (16.7)	1.00		1.00	
** **Yes	17 (20.2)	1.23 (0.68–2.39)	0.460	1.22 (0.63–2.35)	0.553

OR, crude odds ratio; AOR, adjusted odds ratio; CI, confidence interval

^a^ AORs were obtained from a multivariable binary logistic regression model adjusted for energy intake; age; mother’s length of education; frequency of listening to radio

*Statistically significant at p < 0.05

### Diet quality

The mean percentage of the total score for DQI-A was 44.4% ± 7.7%. The average values of the five components of DQI-A were presented on **Table 4**. The mean total DQI-A score and its components, except for DQ, were significantly lower in adolescent girls with anemia. In overweight-obese adolescent girls, only DA was significantly higher than non-overweight-obesity adolescent girls.

**Table 4:** Differences in the total dietary quality indices for adolescents (DQI-A) score among anemic or overweight-obese Indonesian school-going adolescent girls in selected districts of West Java province based on 2-day 24-hour recall.

Component of DQI-A	Score range	Weekday and Weekend Average^a^	Anemia^a^ (n = 329)		Overweight-Obese^a^ (n = 324)	
			**Yes (n = 146)**	**No (183)**	**Yes (n = 57)**	**No (n = 267)**
Dietary Quality (DQ)^b^	−100% to 100%	53.1 (25)	52.5 (25)	53.7 (24)	55.1 (24)	52.7 (25)
Dietary Diversity (DD)^b^	0 to 100%	55.6 (17)	55.6 (14)*	61.1 (11)	61.1 (17)	55.6 (17)
Dietary Adequacy (DA)^c^	0 to 100%	37.0 ± 12.0	34.5 ± 8.2*	39.2 ± 8	39.2 ± 8.9	36.5 ± 8.3*
Dietary Excess (DEx)^b^	0 to 100%	7.0 (7)	5.5 (6)*	7.8 (7)	6.7 (9)	7.1 (7)
Dietary Equilibrium (DE)^c^	0 to 100%	22.6 ± 6.34	21.5 ± 6.6*	23.8 ± 5.8	23.9 ± 7.4	22.4 ± 6.1
**Total DQI-A Score**^c^	33% to 100%	**44.4 ± 7.7**	45.3 ± 8.1*	**42.0 ± 8.9**	**45.1 ± 10.4**	**43.4 ± 8.5**

DQI-A, Dietary Quality Index for Adolescent; DQ, *dietary quality*; DD, *dietary diversity*; DA, *dietary adequacy*

DEx, *dietary excess*; DE, *dietary equilibrium*

^**a**^Based on weekend and weekday average score

^b^Presented in Median (IQR)

^c^Presented in Mean ± SD

*Significantly lower with Mann-Whitney or t-test p<0.05 95%CI

**Tables 5** and **6** present the correlation between dietary quality index, total DQI-A score, and Hb and BAZ score each. Every increase of 1% in total DQI-A score will significantly increase 3.967 g/dL of Hb after adjustment for age, energy, iron intake, highest education of adolescent girls, frequency of reading newspaper, access to internet, and use of social media. Meanwhile, no association was found between the total DQI-A score and the BAZ score.

**Table 5:** Association between dietary quality index and hemoglobin level among school-going adolescent girls in selected districts of West Java (n = 329).

Variable	Hemoglobin^a^ (g/dL)					
	Unadjusted β	P-value	95% CI	Adjusted β^	P-value	95% CI
**Dietary quality index**^**b**^	4.009**	0.000	2.078–5.941	4.390**	0.000	2.396–6.384
Age in year^c^				-0.093	0.105	-0.206–0.020
Frequency of reading newspaper^d^				-0.482	0.012	-0.857 - (-0.108)
Cluster^e^				0.016	0.081	-0.002–0.034

CI, confidence interval

^a^Adjusted for altitude

^b^Diet quality based on mean Total DQI-A score weekend and weekday score

^c^years between 12–19

^d^Not at all compared to Others (Almost every day, at least/less than once a week) as reference

^e^between 17 clusters, ICC 18.4%

^ adjusted for age; frequency of reading newspaper; cluster

** Positively associated with multivariable linear regression model, p <0.01

**Table 6:** Association between dietary quality index and body mass index for age z-score among school-going adolescent girls in selected districts of West Java (n = 324).

Variable	BAZ Score					
	Unadjusted β	P-value	95% CI	Adjusted β^	P-value	95% CI
**Dietary quality index**^a^	0.991	0.111	-0.227–2.209	1.005	0.096	-0.180–2.189
Age in year^b^				-0.150	0.000	-0.217–0.084
Energy^c^ in kcal				0.000	0.068	0.000–0.000
Mother’s length of education^d^				0.071	0.553	-0.164–0.306
Frequency of listening to radio^e^				0.164	0.136	-0.052–0.379

BAZ Score, Body Mass Index for Age Z-score; CI, confidence interval

^a^Diet quality based on mean Total DQI-A score weekend and weekday score

^b^years between 12–19

^c^Use log^10^ energy intake

^d^≤9 compared to >9 (reference)

^e^Not at all compared to Others (Almost every day, at least/less than once a week) as reference

^Adjusted for age; energy intake; mother’s length of education; frequency of listening to radio

**Table 7** shows that the weekday DQI-A score was not significantly different based on eating frequency and dinner skipping. In the other hand, energy intake differs significantly between the eating frequency groups. Less eating frequency on weekday leads to significantly lower energy intake. Moreover, the energy intake tended to be different between adolescent girls who skipped dinner and did not skip dinner on weekdays.

**Table 7:** Comparison between weekday total dietary quality score and energy intake on meal frequencies and dinner skipping among school-going adolescent girls in selected districts of West Java (n = 335).

Variables	Total DQI-A ^a^, median (IQR) %	P-value	Energy intake^b^, median (IQR) kcal	P-value
***Eating Frequency (times/day) Weekday***				
** ***<3*	46.3 (15)	0.084^c^	780 (530)^e^^,^^f^	0.000^c^
** ***3–4*	44.1 (14)		1186 (620)^e^^,^^g^	
** ***>4*	43.3 (16)		1657 (676)^f^^,^^g^	
***Dinner Skipping Weekday***				
** ***No*	44.3 (14)	0.468^d^	1284 (685)	0.051^d^
** ***Yes*	41.4 (17)		1171 (799)	

^a^ Based on weekday total Dietary Quality Index for adolescents (DQI-A) score

^b^ Based on weekday energy intake

^c^ Kruskal-Wallis test significant at p<0.05 95%CI

^d^ Mann-Whitney test significant at p<0.05 95%CI

^e,f,g^ p<0.05 95%CI across groups

### Diet diversity

A percentage of 54.6%, 39.7%, and 5.7% of girls had medium, low, and high dietary diversity, respectively. The breakdown based on food types, anemia status, and weight status are presented in **Table 8**. The mean total dietary score for adolescent girls was 4 out of 9 food categories and the total DDS score between weekend and weekdays were significantly different. Almost all adolescents consumed starch-rich food, a few consumed vegetables with less vitamin-A-rich types than other types of vegetables. Organ meat as a source of iron and folic acid was rarely consumed. About two-thirds of them consumed chicken, meat, and fish, half consumed eggs, legume, nut, and seed, but only approximately 25% consumed dairy products. Adolescent girls with anemia had significantly lower meat, chicken, and fish food group consumption compared to the non-anemic adolescent girls. There was no significant difference in food consumption between the non-overweight-obese and the overweight-obese participants.

**Table 8:** Dietary diversity characteristics based on food types, period of consumption, anemia, and weight status among Indonesian school-going adolescent girls in selected districts of West Java.

Food types	Period of Consumption (N = 335)		Anemia^a^ (N = 329)		Overweight-Obese^a^ (N = 324)	
	Weekend n = 335	Weekday n = 335	No n = 183	Yes n = 152	No n = 267	Yes n = 57
**Starch**, (%)	99.1	100	99.7	99.3	99.4	100
**Green vegetables**, (%)	34.0	28.4	30.1	30.8	31.3	33.3
**Vitamin A fruit and vegetables,** (%)	16.4	14.9	14.5	16.1	15.9	15.8
**Other fruit and vegetables,** (%)	50.1	43.9	47.5	45.6	47.0	50.0
**Organ meat,** (%)	2.4	4.5	4.1	3.1	3.8	3.5
**Meat, chicken, fish,** (%)	74.6	69.6	75.7*	68.2	72.3	71.1
**Eggs,** (%)	49.0	48.4	49.5	48.0	48.3	45.6
**Legumes, nuts, and seeds,** (%)	60.0	62.1	60.9	61	60.7	60.5
**Dairy products,** (%)	30.1	22.1	26.2	25.3	27.7	20.2
**Total Dietary Diversity Score, mean (SD)**	4.13 (1.37)*	3.94 (1.40)*	4.06 (1.06)	3.97 (1.12)	4.1 (1.15)	4.00 (0.85)

DDS, Dietary Diversity Score

a Calculated based on average of weekdays and weekend

* t-test p<0.05 95% CI (0.09–0.15)

Analysis on overall dietary diversity as a grouped score was done. **Tables 9** and **10** present the association between dietary diversity score, anemia, and weight status. There were no association found between dietary diversity and both anemia and overweight-obesity.

**Table 9:** Dietary diversity score as a factor associated to anemia among school-going adolescent girls in selected districts of West Java (n = 329).

Variable	Anemia				
	Yes, n (%)	OR (95% CI)	p–value*	AOR (95% CI)^a^	p–value*
Dietary diversity score^b^					
Low	42 (49.4)	1.39(0.89–2.17)	0.146	1.44 (0.90–2.29)	0.131
Medium-High	110 (44.0)	1.00		1.00	

OR, crude odds ratio; AOR, adjusted odds ratio; CI, confidence interval

^a^ AORs were obtained from a multivariable binary logistic regression model adjusted for age; energy; iron intake; highest education of adolescent girl; frequency of reading newspaper; access to internet; use of social media; cluster (ICC 18.4%)

^b^Low dietary diversity score (DDS) = ≤3.00; Medium = 3.01–6.00; High = 6.01–9

*Statistically significant at p < 0.05

**Table 10:** Dietary quality and diversity as factors associated with weight status among school-going adolescent girls in selected districts of West Java (n = 324).

Variable	Overweight-Obese				
	Yes, n (%)	OR (95% CI)	p–value*	AOR (95% CI)^a^	p–value*
Dietary diversity score^b^					
** **Low	14 (16.5)	0.86 (0.48–1.55)	0.62	0.92 (0.50–1.69)	0.79
** **Medium-High	43 (17.2)	1.00		1.00	

OR, crude odds ratio; AOR, adjusted odds ratio; CI, confidence interval

^a^ AORs were obtained from a multivariable binary logistic regression model adjusted for energy intake; age; mother’s length of education; frequency of listening to radio

^b^Low dietary diversity score (DDS) = ≤3.00; Medium = 3.01–6.00; High = 6.01–9

*Statistically significant at p < 0.05

## Discussion

The current study explored a comprehensive picture of “eating practices” and focused on three constructs: “meal patterning”, “dietary quality” and “dietary diversity” of Indonesian school-going adolescent girls, an approach rarely described by other studies. The findings of the present study revealed that the overall diets of school-going adolescent girls in the study area were poor in quality and low in diversity, and some of the meal patterning, dietary quality, and dietary diversity variables were associated with anemia and overweight-obesity. Eating frequency of 3–4 and >4 times during weekdays in the current study population exhibited greater odds for being anemic. There was also a significant association between skipping dinner on weekdays and being overweight or obese. The energy intake between those who skipped and did not skip dinner on weekdays tended to be different. However, these results need to be interpreted carefully since there was no significant difference in dietary quality regardless of the adolescents’ eating frequency and meal-skipping behavior. Meanwhile, dietary quality, measured by total DQI-A score, was found to be higher in subjects without anemia. No difference was found in the total DQIA score between subjects with an overweight or obesity status and normal BMI, even though dietary adequacy was found to be higher in overweight or obese subjects. Furthermore, linear regression analysis between total DQI-A score and Hb showed a significant increase of 3.967 g/dL of Hb for every 1% increase of total DQI-A score after adjusting for confounders. The overall DDS did not show any significant association with anemia and overweight-obesity, however, the comparison of food consumed by the adolescent girls revealed a significantly higher consumption of meat, chicken, and fish in non-anemic compared to anemic girls.

Our finding, which showed that eating more frequently will result in a significantly higher anemia prevalence among adolescent girls, may be explained by several reasons. One is by the fact that starchy foods may inhibit iron absorption due to the high phytate content [37, 38]. The consumption of starchy food was found to be high in the adolescent girls in this study. Another reason is the fact that frequent consumption of low-diversity meals, as found in the present study, may contribute to low protein and micronutrient intake, both of which play an important role as a carrier and enhancer of iron absorption [39]. Eating frequency may influence energy intake along with nutrient density of iron and folic acid [17]. However, eating frequently does not guarantee nutrient adequacy as it depends on the quality of the diet, as exhibited in the present study: eating more frequently on weekdays does not exhibit greater dietary quality. A possible assumption regarding this result is that dietary quality was poor during the weekdays because the adolescent girls consumed foods from outside of home, such as ones bought from around the schools or leftover meals from the previous days [40]. This condition is likely to be caused by long schooling periods starting early in the morning.

The association between skipping dinner and being overweight-obese was consistent with the result of previous studies, which found significant associations between overweight-obesity and skipping meals [17, 41, 42]. Meal-skipping practice may be associated with perception of body image and weight loss among adolescent girls [42] and was a common habit among the adolescents, causing not only difficulties in achieving nutrient adequacy [43], but also higher consumption of energy, carbohydrate, and vitamin K [17]. Adolescent girls who skipped meals were more likely to eat snacks, causing excessive energy intake [44]. In the present study, the energy intake between the girls who skipped dinner and had dinner on weekdays was not significantly different. Therefore, it was reasonable that in the present study, skipping dinner led to higher odds of being overweight-obese.

Significant difference in dietary quality between anemic and non-anemic adolescent girls in the current study is also consistent with a previous study in Indonesia, which found a significant association between dietary quality and microcytic-hypochromic anemia [10]. Secondary analysis between the DQIA score and Hb value also showed a positive association, supporting the premise that higher dietary quality may lead to higher hemoglobin concentration, and therefore prevents anemia.

DQIA score was not significantly different between overweight-obese and normal-weight individuals. Adolescent girls with overweight or obesity were reported to have higher DA scores compared to girls with normal BMI, however, no difference in DEx score was found, showing no significant excessive consumption among subjects with overweight-obesity compared to subjects with normal BMI. The result was in contrast with previous studies that found an association between low dietary quality and overweight-obesity [24, 45]. The relatively poor overall dietary quality could be one of the possible explanations to the current result.

Although dietary diversity component was included as one of the total DQI-A components, this study more specifically used DDS tool to assess dietary diversity in reproductive-age women. DDS food grouping differentiates protein sources into meat, chicken, fish, organ meat, and eggs [33], which are grouped into a single protein source group in the DD of DQI-A [34]. Dietary diversity by DDS found that adolescent girls with anemia consumed significantly less meat, chicken, and fish. However, the difference in overall dietary diversity between anemic and non-anemic adolescent girls as well as between overweight-obese and normal-weight adolescent girls, represented by total DDS score, did not show significant results. Dietary diversity resulted in an adequacy of macro- and micronutrient intake [46] and can be used as a standard for assessing micronutrient adequacy. Higher diversity of food is associated with higher nutrient intake of all vitamins especially vitamin B12 and C [47]. However, as with our study, another study in Nigeria also did not find any association between dietary diversity and anemia [23] or overweight/obesity among adolescent girls [48]. The difference in results of DDS and DD of DQI-A components was possibly due to the different methods of food grouping.

The present study’s strength is that we assessed both dietary quality and quantity of adolescent girls, wherein quantity was assessed by using a neutral definition, based on minimum kcal and spacing time between meals. This approach is more consistent and standardized compared to approaches used in other studies [17]. An example of other alternative approaches is participant-identified definition, which used the participant herself for defining one eating occasion, such as in the study done by Howarth et al [49]. That definition is prone to bias due to the different perspectives about what constitutes an eating event. The dietary quality index used in the mentioned study is also less variable as it is not based on the amount of nutrients consumed. The dietary quality indices described in our study (using DQI-A) are especially made for adolescent populations. In addition, the dietary quality of the collected data has been appropriately assessed by taking into consideration the quantity of foods consumed even in the condition where the use of food composition tables is limited in the analysis.

This study also treated the data collection and analysis very carefully by pre-testing the method, standardizing the portions using colored photographs, reducing operator bias by assigning the same person to take the repeated recalls per subject, and using local standards of food composition table to calculate the nutrient contents of local foods and snacks. The DQI-A used to assess dietary quality in the present study is a valid scoring system used specifically for adolescents. The DDS used for the assessment in this study was adapted from the most recent guidelines for women of reproductive age to be as close as possible to the studied age range. Anthropometric and Hb concentration measurements were also done by professionally trained personnel. Therefore, the values and findings presented in this study were less likely to have been found by chance.

No studies have assessed comprehensive meal patterning characteristics (frequency, skipping, and spacing) to date. Frequency of eating occasions and meal skipping are often only assessed individually for defining meal pattern. However, the ways by which adolescent girls consume their food are unique, often combining whole meals with snacks, and in many occasions eating out with their peers, thus following the eating patterns of their peers that may be lower in nutrient content. Therefore, the findings of this study provide better understanding of the ways in which different meal patterns make an impact on dietary quality, which might help elucidate important diet–disease relationships. Moreover, a meal-pattern-based approach could complement current dietary advice, which in some countries currently uses a food-based framework, to assist adolescent girls with simple messages in achieving their recommended daily intake of food and nutrients. That is, dietary advice in the context of specific meal patterning and dietary examples could help the girls with their daily meal preparation and therefore be a more practical and salient way to assist them to follow dietary guidelines [17].

Some limitations of the present study include possible bias and error in the data collection and analysis, despite the efforts to minimize them. The data collection used 2-day repeated 24-hour recall which relies on the participants’ memory. Considering this method may be prone to under/over reporting of meals and under/overestimating of portions, we controlled the bias by having the same assessor for 2 collection days and standardized the portion size aided with visual tools. However, not all meals recalled were informed in detail, which may have affected the food grouping and DQI-A scoring. In addition, despite the reliability of HemoCue^TM^ compared to other portable Hb measurement devices, the formal laboratory venous sampling is still the gold standard for Hb measurement [29].

Moreover, although the method included here of assessing meal patterning, dietary quality and diversity from 2-day repeated 24-hour recall was useful in identifying some dietary concerns, it does not clearly define the long-term pattern of these variables. This cross-sectional study also cannot represent causality or reverse causality of the variables. Multiple factors are known to have an impact on anemia, including intestinal infections, thalassemia, and chronic diseases [50, 51], so it is not plausible to differentiate whether the anemia is caused by nutrient deficiency or other causes based solely on the indicators measured in this study. Therefore, further studies are needed to draw better conclusions, accounting for other factors as potential confounders.

## Conclusions

We observed a higher quality in the diet of non-anemic adolescent girls compared to that of anemic girls. Meanwhile, an increment of total DQI-A score was associated with a higher Hb concentration. Although no associations were found between dietary quality and overweight-obesity, overweight-obesity suggested to be affected by meal patterning. Further study is needed to explain how the trend of eating more frequently could lead to eating more low-quality and less diverse foods. Choosing sufficient, non-excessive meal patterning along with improving dietary quality and diversity in adolescent girls is important to support the prevention of anemia and overweight-obesity in the growing age group. This study, along with findings from further research, should be considered when advising the details of proper meal patterning for adolescent girls and for encouraging regular consumption of nutrient-rich foods. Nutrition education for school-age adolescent girls is also encouraged to promote balanced nutrition and dietary diversity.

## Supporting information

S1 Data (XLSX)
